# Associations between inflammatory gene polymorphisms (TNF-α 308G/A, TNF-α 238G/A, TNF-β 252A/G, TGF-β1 29T/C, IL-6 174G/C and IL-10 1082A/G) and susceptibility to osteosarcoma: a meta-analysis and literature review

**DOI:** 10.18632/oncotarget.18813

**Published:** 2017-06-29

**Authors:** Yanhua Jiang, Xiandi Wang, Yi Cheng, Jun Peng, Jiwei Xiao, Dingbo Tang, Ying Yi

**Affiliations:** ^1^ Department of Orthopedics, Sichuan Cancer Hospital, Chengdu, China; ^2^ Department of Orthopedics, Huashan Hospital, Fudan University, Shanghai, China

**Keywords:** osteosarcoma, inflammatory gene, polymorphism, meta-analysis

## Abstract

Associations between inflammatory gene polymorphisms (TNF-α 308G/A, TNF-α 238G/A, TNF-β 252A/G, TGF-β1 29T/C, IL-6 174G/C and IL-10 1082A/G) and osteosarcoma (OS) risk remain unclear. We conducted a systematic search to retrieve studies that investigated associations between inflammatory gene polymorphisms and OS risk. Nine studies that met the inclusion criteria were finally recruited in this meta-analysis. Overall, there was a significant association between TNF-α 308G/A, IL-10 1082A/G and OS risk, while there was no significant association between TNF-α 238G/A, TNF-β 252A/G and IL-6 174G/C and OS risk. Our subgroup analysis showed a significant association between IL-6 174G/C and IL-10 1082A/G and OS risk in Asians, while no such significant correlation was observed with TNF-α 308G/A, TNF-α 238G/A, TNF-β 252A/G and TGF-β1 29T/C polymorphisms. In Caucasians, there was a significant association between TNF-α 238G/A and the decreased incidence of OS. In conclusion, inflammatory gene polymorphisms play a key role in the occurrence and progression of OS. IL-6 174G/C polymorphism was obviously associated with OS risk in Asians, while TNF-α 238G/A polymorphism seemed to be associated with the decreased susceptibility to OS in Caucasians as Altman and Bland test indicated. Although controversial results were observed between IL-10 1082A/G and OS risk in Asians and Caucasians, it is difficult to make a definite conclusion about the role of IL-10 1082A/G polymorphism in the etiology of OS because our Altman and Bland test showed no good evidence to support a different effect in Asians and Caucasians.

## INTRODUCTION

Osteosarcoma (OS) is one of the most common primary malignant bone tumors probably due to repeated production of osteoid by malignant cells [1, 2]. OS is a high-grade malignant mesenchymal tumor in the bone tissue that often leads to high disability and mortality in both children and adults, causing great burdens to families and society [1, 3, 4]. According to a survey conducted by the Surveillance, Epidemiology, and End Results (SEER), about 3010 people were diagnosed with primary malignant tumors of bone and joints in the United States in 2013 [5]. In China, OS is also the most common primary malignant bone and soft tissue tumor. According to the database of Beijing Jishuitan Hospital (one of the most famous orthopedics hospitals in China) [6], OS is the most common histologic type of bone tumors, accounting for 22.8% of all bone tumors, followed by giant cell tumor (16.7%), osteochondroma (9.1%), and primary chondrosarcoma (5.6%).

The etiology of OS is complex involving multiple factors, but its actual pathogenesis remains unclear [1, 2, 7, 8]. Abnormal bone growth is reported to be significantly associated with the development and progression of OS [9], and diverse strategies have been attempted to check the progression of OS during the rapid stage of bone growth [10]. Environmental factors have also been found to be associated with OS risk [11, 12]. In addition, age [13], gender [14], ethnicity [15], fluorinated drinking water [16], height [17, 18], and other factors [1] have also been implied as risk factors of OS.

In addition to the above-mentioned risk factors, more studies have verified the importance of genes and gene polymorphisms in the etiology, development and complexity of OS [1, 4, 8]. Copy number gains at chromosomes 1p, 1q, 6p, 8q and 17p, and copy number losses at chromosomes 3q, 6q, 9, 10, 13, 17p and 18q have been reported by many research groups, including the systematic review regarding the role of genes in the etiology of OS conducted by Martin et al. [8]. In addition, more gene polymorphisms have been found to play a key role in the etiology and development of OS, highlighting the importance of gene polymorphisms in this disease [19–22].

Inflammation has been increasingly recognized as an important factor in the etiology and development of cancers, for many studies have demonstrated that inflammatory cells play an important role in the tumor microenvironment, where they foster tumor cell proliferation, survival and migration [23–25] and that gene polymorphisms also play important roles in various cancers and diseases including OS [26–28]. Zhao et al. [29] showed that the AA genotype of TNF-α-308G/A locus of TNF-α gene was a risk factor for OS. However, Oliveira et al. [26] reported that there was no significant association between them. The result of a previous meta-analysis performed to detect the correlation of TNF-α 308G/A, TNF-β (Tumor Necrosis Factor-β) 252A/G and TGF-β1 (Transforming Growth Factor-β1) 29T/C polymorphisms with OS risk [30] showed that TGF-β1 29T/C variants were significantly associated with OS while no association was found between TNF-α -308G/A or TNF-β 252A/G polymorphism and OS risk. But as their meta-analysis only focused on the association of TNF-α 308G/A, TNF-β 252A/G and TGF-β1 29T/C polymorphisms with OS susceptibility, whether other gene polymorphisms such as IL-6 (Interleukin 6), IL-10 (Interleukin 10) and TNF-α 238G/A play a key role in the etiology and development of OS remains unclear. Although increased numbers of inflammatory gene polymorphisms have been found to be associated with susceptibility to OS [26, 31–34], no meta-analysis has been conducted to explore the associations between these novel inflammatory gene polymorphisms and OS risk. The aim of our meta-analysis is to explore the associations of inflammatory gene polymorphisms (TNF-α 308G/A, TNF-α 238G/A, TNF-β 252A/G, TGF-β1 29T/C, IL-6 174G/C and IL-10 1082A/G) with OS risk.

## MATERIALS AND METHODS

### Literature search

We conducted a systematic online search using ‘PubMed’, ‘EMBASE’, ‘Web of Science’, ‘the Cochrane Library’, ‘Science Direct’, ‘Karger’, ‘Wiley Online Library’ and ‘Springer’ and ‘China WeiPu Library’ to identify case-control studies that investigated the relationship between inflammatory gene polymorphisms and susceptibility to OS. First, the following search terms were used to find out how many inflammatory gene polymorphisms were studied: (‘osteosarcoma’ OR ‘OS’ OR ‘osteosarcoma tumor’) AND (‘polymorphism’ OR ‘single nucleotide polymorphism’ OR ‘SNP’ OR ‘variation’), and then, one-by-one screening was performed by two authors. We found that inflammatory gene polymorphisms TNF-α, TNF-β, TGF-β1, IL-1, IL-6, IL-8, IL-10, IL-12 and IL-16 were studied in case-control studies. Next, we used search terms one-by-one to find out whether there were sufficient articles that reported at least one of the gene polymorphisms identified in our previous search stage. The following search terms were used to find out the eligible studies regarding the association between TNF polymorphisms and OS risk: (‘TNF’ OR ‘Tumor Necrosis Factor) AND (‘osteosarcoma’ OR ‘OS’ OR ‘osteosarcoma tumor’) AND (‘polymorphism’ OR ‘single nucleotide polymorphism’ OR ‘SNP’ OR ‘variation’). The search terms, (‘TGF’ OR ‘Transforming Growth Factor’) AND (‘osteosarcoma’ OR ‘OS’ OR ‘osteosarcoma tumor’) AND (‘polymorphism’ OR ‘single nucleotide polymorphism’ OR ‘SNP’ OR ‘variation’) were used to find the associations between TGF and OS risk. As to IL-6, the following search terms were used: (‘IL 6’ OR ‘Interleukin 6’) AND (‘osteosarcoma’ OR ‘OS’ OR ‘osteosarcoma tumor’) AND (‘polymorphism’ OR ‘single nucleotide polymorphism’ OR ‘SNP’ OR ‘variation’). As to IL-10, the following search terms were used: (‘IL 10’ OR ‘Interleukin 10’) AND (‘osteosarcoma’ OR ‘OS’ OR ‘osteosarcoma tumor’) AND (‘polymorphism’ OR ‘single nucleotide polymorphism’ OR ‘SNP’ OR ‘variation’). And then, these studies were further selected. Furthermore, no language restrictions were applied. Secondary searches of the unpublished literature were conducted by searching the reference lists of the selected studies, reviews and conference reports. Reviews and comments were also examined to further identify eligible studies.

### Inclusion and exclusion criteria

The inclusion criteria of our meta-analysis were as follows: (1) case-control studies; (2) evaluation of OS risk and at least one of the identified inflammatory gene polymorphisms were reported; (3) having detailed genotype frequency or numbers of alleles and genotypes between cases and controls or effect size reported or estimated from the articles. The exclusion criteria were: (1) reviews or case reports that were not case-control studies; (2) no available data reported; (3) duplicated reports.

### Data extraction

Data from the eligible studies were extracted according to the inclusion and exclusion criteria by two authors, and a consensus was reached. For each study, the following data were collected: author list, year of publication, ethnicity, sample size, alleles, genotype of each gene polymorphism, and HWE (Hardy-Weinberg Equilibrium). Incomplete data were also obtained by contacting the respective corresponding author.

### Data synthesis and statistical analysis

Odds ratios (OR) and 95% confidence interval (CI) were calculated to evaluate the association between inflammatory gene polymorphisms and OS. An allele contrast model (A vs. G), heterozygote model (AG vs. GG), homozygote model (AA vs. GG), dominant (AA/AG vs. GG), and recessive (AA vs. AG/GG) model were used for TNF-α 308G/A and TNF-α 238G/A polymorphism. An allele contrast model (G vs. A), heterozygote model (AG vs. AA), homozygote model (GG vs. AA), dominant (GG/AG vs. AA), and recessive (GG vs. AG/AA) model were used for TNF-β 252A/G and IL-10 1082A/G polymorphism. An allele contrast model (C vs. T), heterozygote model (TC vs. TT), homozygote model (CC vs. TT), dominant (CC/TC vs. TT), and recessive (CC vs. TC/TT) model were used for TGF-β1 29T/C polymorphism. And the strength of association between IL-6 174G/C polymorphism and OS susceptibility was evaluated by OR and 95% CI according to allele contrast model (C vs. G), heterozygote model (GC vs. GG), homozygote model (CC vs. GG), dominant (GC/CC vs. GG), and recessive (CC vs. GC/GG) model.

The assumption of heterogeneity was verified by a chi-squared-based Q statistical test and quantified by I^2^ metric value. ORs were pooled by random effect model across the study, which considers both within-study and between-study variation.

Sensitivity analysis was performed to assess the impact of each study on the combined effect of the present meta-analysis, and subgroup analysis was performed according to the ethnicity of the study populations. Altman and Bland test [35] was conducted to evaluate the difference between Asians and Caucasians. The ratio of relative risk (RRR) with 95% CI and z score were used to assess the interaction. HWE was calculated in control population to evaluate the quality of the data by using chi-square test. All meta-analyses were conducted using Stata 12.0 software (StataCorp, College Station, TX, USA) and a P value below 0.05 was considered statistically significant.

## RESULTS

**Figure 1:**
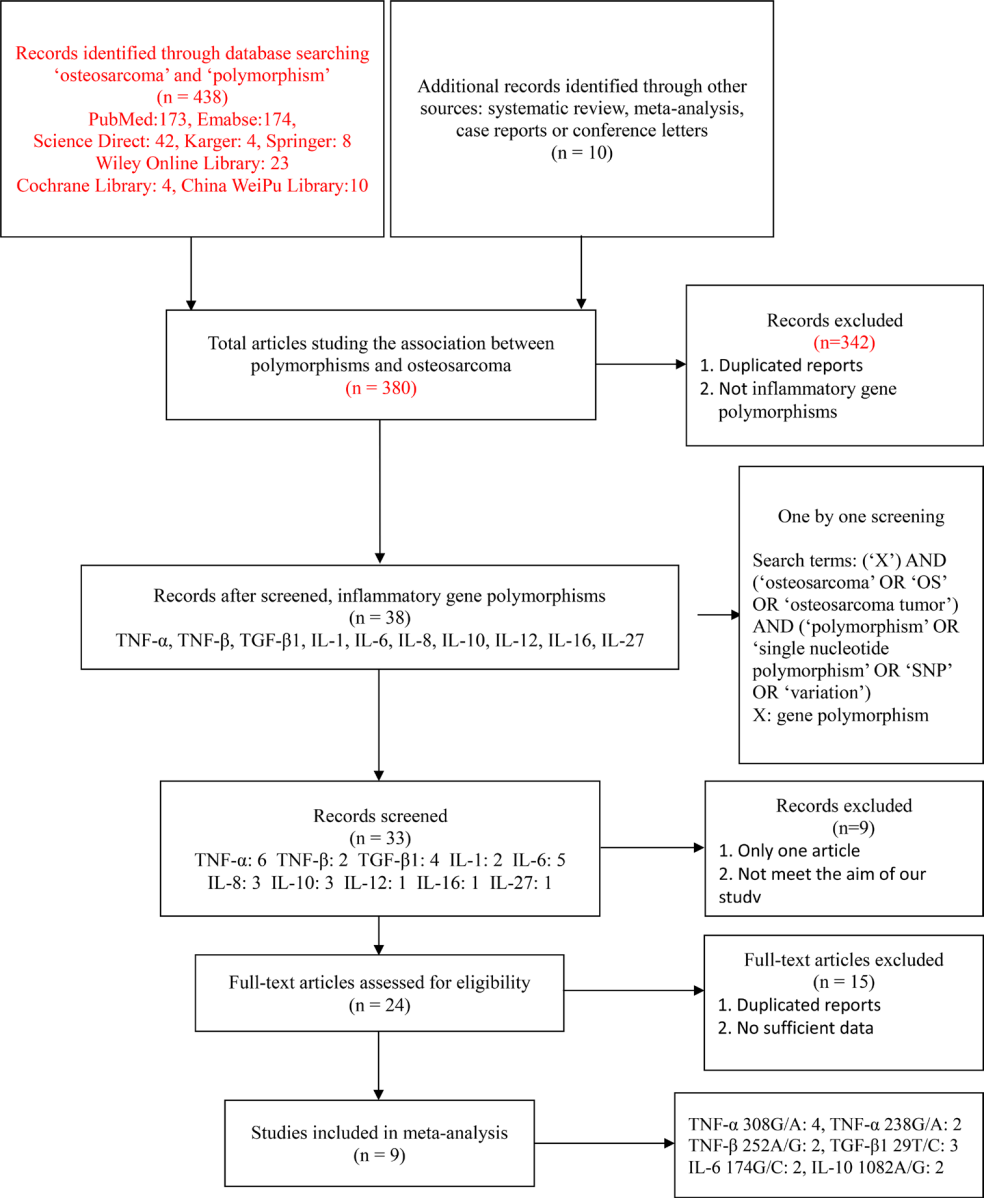
The study selection and inclusion process

A total of nine studies [26, 29, 32, 33, 36–40] with 1119 cases and 1358 controls were recruited in this meta-analysis. The process of study selection and the inclusion process are shown in Figure 1. Four studies [26, 29, 36, 37] focused on the association between TNF-α 308G/A polymorphism and OS risk, among which two studies [29, 36] also reported both alleles and genotypes of TNF-α 238G/A polymorphism. Oliveira et al. [26] and Xie et al. [37] studied the relationship between TNF-β 252A/G and susceptibility to OS. Ma et al. [38], Xu et al. [39] and Wu et al. [40] reported the alleles and genotypes of TGF-β1 29T/C polymorphism. Oliveira et al. [26] reported the alleles and genotypes of IL-6 174G/C and IL-10 1082A/G polymorphism, and these two polymorphisms were also studied by Qi et al. [32] and Cui et al. [33]. Furthermore, seven studies [29, 32, 33, 37–40] were performed in Asian populations, and the other two studies [26, 36] were conducted in Caucasian populations. In addition, all these studies had complied with HWE. The general characteristics of the recruited studies are summarized in Table 1.

**Table 1:** General characteristics of studies included in the meta-analysis

Author	Year	Ethnicity	Sample size		TNF-α 308G/A Case		TNF-α 308G/A Control		HWE	TNF-α 238G/A Case		TNF-α 238G/A Control			HWE
			Case	Control	G/A	AA/AG/GG	G/A	AA/AG/GG		G/A	AA/AG/GG	G/A	AA/AG/GG		
Patio-Garcia A et al.	2000	Caucasian	63	111	112/14	0/14/49	194/28	0/28/83	0.13	124/2	0/2/61	204/18	0/18/93		0.35
Oliveira ID et al.	2007	Caucasian	80	160	129/31	6/19/55	285/35	5/25/130	0.15						
Xie et al.	2008	Asian	52	60	103/1	0/1/51	110/10	1/8/51	0.32						
Zhao et al.	2015	Asian	80	99	94/66	16/34/30	138/60	6/48/45	0.14	140/20	2/16/62	172/26	1/24/74		0.53

					TNF-β 252A/G Case		TNF-β 252A/G Control		HWE	TGF-β1 29T/C Case		TGF-β1 29T/C Control			HWE
					A/G	AA/AG/GG	A/G	AA/AG/GG		T/C	TT/TC/CC	T/C		TT/TC/CC	
Oliveira ID et al.	2007	Caucasian	80	160	55/105	9/37/34	100/220	17/66/77	0.61						
Xie et al.	2008	Asian	52	60	57/47	16/25/11	67/53	20/27/13	0.50						
Ma et al.	2010	Asian	42	100						30/54	6/18/18	91/109		16/59/25	0.06
Xu et al.	2014	Asian	202	216						186/218	42/102/58	242/190		66/110/40	0.62
Wu et al.	2015	Asian	124	136						149/99	52/45/27	133/139		37/59/40	0.12

					IL-6 174G/C Case		IL-6 174G/C Control		HWE	IL-10 1082A/G Case		IL-10 1082A/G Control			HWE
					G/C	GG/GC/CC	G/C	GG/GC/CC		A/G	AA/AG/GG	A/G		AA/AG/GG	
Oliveira ID et al.	2007	Caucasian	80	160	50/108	8/34/37	87/231	12/63/84	0.97	56/104	10/3634	125/195		25/75/60	
Qi et al.	2016	Asian	216	216	271/161	94/83/39	322/110	130/62/24	0.30						
Cui et al.	2016	Asian								351/169	139/73/48	386/134		155/76/29	0.35

### Meta-analysis results

In the overall populations, TNF-α 308G/A and IL-10 1082A/G polymorphisms were significantly associated with the increased susceptibility to OS (TNF-α 308G/A: AA vs. GG: OR = 2.99, 95% CI = 1.34–6.68, *P* = 0.007; AA vs. AG/GG: OR = 2.90, 95% CI = 1.37–6.13, *P* = 0.005; IL-10 1082A/G (G vs. A: OR = 1.32, 95% CI = 1.06–1.65, *P* = 0.014; GG vs. AA: OR = 1.72, 95% CI = 1.11–2.67, *P* = 0.016; GG vs. AA/AG: OR = 1.52, 95% CI = 1.05–2.20, *P* = 0.028), while no significant association was found between TNF-α 238G/A, TNF-β 252A/G and IL-6 174G/C and OS risk (all *P* > 0.05) (Tables 2 and 3).

**Table 2:** Results of genetic models for TNF-α 308G/A, TNF-α 238G/A, TGF-β 252A/G and TGF-β1 29T/C polymorphisms and osteosarcoma

Comparison	*N*	Test of association			Test of heterogeneity		Altman and Bland test		
		OR	95% CI	*P* value	*P* value	I^2^ (%)	Model	Ratio of relative risk (RRR), 95% CI	Test of interaction (z score)
TNF-α 308G/A (rs1800629)									
Caucasian A *vs.* G AA *vs.* GG AG *vs.* GG AA/AG *vs.* GG AA *vs.* AG/GG	2	1.34 2.84 1.25 1.32 2.51	0.60–2.98 0.83–9.68 0.60–2.61 0.58–3.02 0.74–8.50	0.470 0.096 0.551 0.507 0.138	0.064 - -0.139 0.084 -	70.9 - 54.4 66.5 -	A *vs.* G: AA *vs.* GG: AG *vs.* GG: AA/AG *vs.* GG: AA *vs.* AG/GG:	2.68 (0.15–47.4) 1.52 (0.12–19.61) 2.72 (0.30–24.3) 2.75 (0.20–38.02) 1.23 (0.11–13.39)	0.67 0.32 0.89 0.75 0.17
Asian A *vs.* G AA *vs.* GG AG *vs.* GG AA/AG *vs.* GG AA *vs.* AG/GG	2	0.50 1.87 0.46 0.48 2.04	0.03–7.46 0.20–17.80 0.06–3.71 0.04–5.86 0.26–15.75	0.616 0.585 0.468 0.565 0.494	0.010 0.149 0053 0.020 0.175	84.9 52 73.2 81.4 45.7			
Overall A *vs.* G AA *vs.* GG AG *vs.* GG AA/AG *vs.* GG AA *vs.* AG/GG	4	1.18 2.99 1.00 1.09 2.90	0.63–2.21 1.34–6.68 0.53–1.87 0.55–2.15 1.37–6.13	0.599 0.007 0.998 0.804 0.005	0.019 0.351 0.082 0.036 0.383	69.9 4.4 55.2 64.8 0			
TNF-α 238G/A (rs361525)								
Caucasian A *vs.* G AA *vs.* GG AG *vs.* GG AA/AG *vs.* GG AA *vs.* AG/GG	1	**0.18** 1.52 **0.17** **0.17** 1.75	**0.04–0.80** 0.09–24.69 **0.04–0.76** **0.04–0.76** 0.11–28.46	**0.024** 0.770 **0.020** **0.020** 0.694	- - - - -	- - - - -	A *vs.* G: AA *vs.* GG: AG *vs.* GG: AA/AG *vs.* GG: AA *vs.* AG/GG:	0.19 (0.04–0.96) 0.64 (0.02–26.01) 0.21 (0.04–1.09) 0.20 (0.04–1.00) 0.70 (0.02–27.89)	-2.01 -0.24 -1.85 -2.00 -0.19
Asian A *vs.* G AA *vs.* GG AG *vs.* GG AA/AG *vs.* GG AA *vs.* AG/GG	1	0.95 2.39 0.80 0.86 2.51	0.51–1.76 0.21–26.95 0.39–1.63 0.43–1.72 0.22–28.23	0.859 0.482 0.532 0.668 0.455	- - - - -	- - - - -			
Overall A *vs.* G AA *vs.* GG AG *vs.* GG AA/AG *vs.* GG AA *vs.* AG/GG	2	0.48 1.96 0.42 0.44 2.15	0.09–2.41 0.32–12.24 0.09–1.93 0.09–2.17 0.35–13.37	0.370 0.470 0.266 0.311 0.411	0.040 0.810 0.062 0.049 0.848	76.3 0 71.2 74.2 0			
TNF-β 252A/G (rs909253)								
Caucasian G *vs.* A GG *vs.* AA AG *vs.* AA GG/AG *vs.* AA GG *vs.* AG/AA	1	0.87 0.83 1.06 0.94 0.80	0.58–1.30 0.34–2.06 0.43–2.61 0.40–2.21 0.46–1.37	0.490 0.694 0.901 0.883 0.410	- - - - -	- - - - -	G *vs.* A GG *vs.* AA AG *vs.* AA GG/AG *vs.* AA GG *vs.* AG/AA	0.84 (0.43–1.63) 0.78 (0.20–3.11) 0.91 (0.26–3.17) 0.83 (0.26–2.67) 0.82 (0.29–2.38)	-0.52 -0.35 -0.14 -0.31 -0.36
Asian G *vs.* A GG *vs.* AA AG *vs.* AA GG/AG *vs.* AA GG *vs.* AG/AA	1	1.04 1.06 1.16 1.13 0.97	0.61–1.77 0.37–2.99 0.49–2.72 0.51–2.50 0.39–2.40	0.878 0.916 0.737 0.772 0.947	- - - -	- - - - -			
Overall G *vs.* A GG *vs.* AA AG *vs.* AA GG/AG *vs.* AA GG *vs.* AG/AA	2	0.93 0.92 1.11 1.03 0.84	0.67–1.28 0.47–1.83 0.60–2.06 0.58–1.85 0.53–1.34	0.649 0.820 0.742 0.911 0.459	0.589 0.735 0.888 0.760 0.715	0 0 0 0 0			
TGF-β1 29T/C (rs1800470)								
Asian C *vs.* T CC *vs.* TT TC *vs.* TT CC/TC *vs.* TT CC *vs.* TC/TT	3	1.12 1.25 0.89 0.99 1.36	0.62–2.02 0.42–3.76 0.44–1.79 0.43–2.29 0.66–2.79	0.717 0.687 0.735 0.982 0.399	< 0.001 0.001 0.032 0.003 0.011	87.2 85.1 71.1 82.4 77.9			

**Table 3:** Results of genetic models for IL-6 174G/C and IL-10 1082A/G polymorphisms and osteosarcoma

Comparison	*N*	Test of association			Test of heterogeneity		Altman and Bland test		
		OR	95% CI	*P* value	*P* value	I^2^ (%)	Model	Ratio of relative risk (RRR), 95% CI	Test of interaction (z score)
IL-6 174G/C (rs1800795)									
Caucasian C *vs.* G CC *vs.* GG GC *vs.* GG CC/GC *vs.* GG CC *vs.* GC/GG	1	1.21 0.66 0.81 0.72 0.79	0.57*−*2.54 0.25*−*1.75 0.30*−*2.17 0.28*−*1.85 0.46*−*1.35	0.331 0.405 0.675 0.501 0.384	- - - - -	- - - - -	C *vs.* G CC *vs.* GG GC *vs.* GG CC/GC *vs.* GG CC *vs.* GC/GG	0.70 (0.31*−*1.55) 0.29 (0.09*−*0.91) 0.44 (0.15*−*1.28) 0.37 (0.13*−*1.02) 0.45 (0.21*−*0.97)	−0.89 *−*2.13 *−*1.50 *−*1.93 *−*2.04
Asian C *vs.* G CC *vs.* GG GC *vs.* GG CC/GC *vs.* GG CC *vs.* GC/GG	1	**1.74** **2.25** **1.85** **1.96** **1.76**	**1.30***−***2.33** **1.27***−***3.99** **1.21***−***2.83** **1.34***−***2.88** **1.02***−***3.05**	**< 0.001** **0.006** **0.004** **0.001** **0.043**	- - - - -	- - - - -			
Overall C *vs.* G CC *vs.* GG GC *vs.* GG CC/GC *vs.* GG CC *vs.* GC/GG	2	1.21 1.30 1.39 1.31 1.18	0.57*−*2.54 0.39*−*4.29 0.64*−*3.00 0.50*−*3.42 0.53*−*2.59	0.619 0.665 0.406 0.578 0.688	0.003 0.034 0.131 0.054 0.040	88.4 77.8 56.1 73.1 76.3			
IL-10 1082A/G (rs1800896)									
Caucasian G *vs.* A GG *vs.* AA AG *vs.* AA AG/GG *vs.* AA GG *vs.* AA/AG	1	1.19 1.42 1.20 1.30 1.23	0.80*−*1.77 0.61*−*3.30 0.52*−*2.76 0.59*−*2.85 0.71*−*2.13	0.387 0.419 0.668 0.519 0.455	- - - - -	- - - - -	G *vs.* A GG *vs.* AA AG *vs.* AA AG/GG *vs.* AA GG *vs.* AA/AG	0.86 (0.53*−*1.38) 0.77 (0.29*−*2.06) 1.12 (0.45*−*2.82) 1.01 (0.43*−*2.38) 0.68 (0.32*−*1.43)	*−*0.64 *−*0.52 0.24 0.02 *−*1.01
Asian G *vs.* A GG *vs.* AA AG *vs.* AA AG/GG *vs.* AA GG *vs.* AA/AG	1	1.39 1.85 1.07 1.29 1.80	1.06*−*1.81 1.10*−*3.09 0.72*−*1.59 0.91*−*1.82 1.10*−*2.97	0.017 0.020 0.733 0.157 0.020	- - - - -	- - - - -			
Overall G *vs.* A GG *vs.* AA AG *vs.* AA AG/GG *vs.* AA GG *vs.* AA/AG	2	1.32 1.72 1.09 1.29 1.52	1.06*−*1.65 1.11*−*2.67 0.77*−*1.56 0.94*−*1.77 1.05*−*2.20	0.014 0.016 0.623 0.120 0.028	0.531 0.600 0.809 0.984 0.312	0 0 0 0 2.2			

Our subgroup analysis showed that there was no significant association between TNF-α 308G/A and TNF-β 252A/G polymorphisms and susceptibility to OS in either Asian or Caucasian populations (both *P* > 0.05) (Table 2). Altman and Bland test showed consistency of our subgroup results regarding the role of TNF-α 308G/A and TNF-β 252A/G polymorphisms in OS etiology (Table 2).

It was found in our meta-analysis that TNF-α 238G/A polymorphism was a protective contributor to OS susceptibility in Caucasian populations (A vs. G: OR = 0.18, 95% CI = 0.04–0.80, *P* = 0.024; AG vs. GG: OR = 0.17, 95% CI = 0.04–0.76, *P* = 0.020; AA/AG vs. GG: OR = 0.17, 95% CI = 0.04–0.76, *P* = 0.020, Table 2). However, we could not find any association between TNF-α 238G/A polymorphism and susceptibility to OS in the Asian populations (Table 2), suggesting that TNF-α 238G/A polymorphism might play a different role in the etiology of OS in different ethnicities, as our Altman and Bland test showed that there was good evidence to support a different effect in Asians and Caucasians [A vs. G: ratio of relative risk (95% CI) = 0.19 (0.04–0.96), z score = -2.01; AA/AG vs. GG: ratio of relative risk (95% CI) = 0.20 (0.04–1.00), z score = −2.00]. All these data are shown in Table 2.

As shown in Table 3, although no significant association was observed between IL-6 174G/C polymorphism and OS risk in the overall and Caucasian populations (both *P* > 0.05), IL-6 174G/C mutation might increase the risk of OS in Asian populations (C vs. G: OR = 1.74, 95% CI = 1.30–2.33, *P* < 0.001; CC vs. GG: OR = 2.25, 95% CI = 1.27–3.99, *P* = 0.006; GC vs. GG: OR = 1.85, 95% CI = 1.21–2.83, *P* = 0.004; CC/GC vs. GG: OR = 1.96, 95% CI = 1.34–2.88, *P* = 0.001; CC vs. GC/GG: OR = 1.76, 95% CI = 1.02–3.05, *P* = 0.043). This controversial result might be attributed to the different genetic backgrounds in Asians and Caucasians, as shown by the results of Altman and Bland test [CC vs. GG: ratio of relative risk (95% CI) = 0.29 (0.09–0.91), z score = −2.13; CC vs. GC/GG: ratio of relative risk (95% CI) = 0.45 (0.21–0.97), z score = −2.04], while other factors such as the limited number of included studies or the small number of included cases in individual studies could not be ignored.

A significant association was also observed between IL-10 1082A/G polymorphism and OS risk in Asian populations (G vs. A: OR = 1.39, 95% CI = 1.06–1.81, *P* = 0.017; GG vs. AA: OR = 1.85, 95% CI = 1.10–3.09, *P* = 0.020; GG vs. AA/AG: OR = 1.80, 95% CI = 1.10–2.97, *P* = 0.020, Table 3); however, we did not find any significant association in Caucasian populations (all *P* > 0.05) (Table 3). We could hardly make a certain conclusion regarding the role of IL-10 1082A/G polymorphism in the etiology of OS, for our Altman and Bland test showed that there was no good evidence to support a different effect in Asians and Caucasians [G vs. A: ratio of relative risk (95% CI) = 0.86 (0.53–1.38), z score = −0.64; CC vs. GG vs. AA: ratio of relative risk (95% CI) = 0.77 (0.29–2.06), z score = −0.52; AG vs. AA: ratio of relative risk (95% CI) = 1.12 (0.45–2.82), z score = 0.24; AG/GG vs. AA: ratio of relative risk (95% CI) = 1.01 (0.43–2.38), z score = 0.02; GG vs. AA/AG: ratio of relative risk (95% CI) = 0.68 (0.32–1.43), z score = −1.01].

As to TGF-β1 29T/C polymorphism, no significant association with OS was observed in Asian populations (*P* > 0.05) (Table 2). As no study reported the association between TGF-β1 29T/C polymorphism and OS risk, whether it plays a key role in Caucasian populations remains unclear.

### Sensitivity analysis and publication bias

A leave-one-out analysis was performed to estimate the sensitivity of our meta-analysis. Any single study could be omitted, without any effect on the overall statistical significance, indicating that the results are stable. Furthermore, we did not assess publication bias because the number of studies analyzed in each polymorphism is smaller than 10.

## DISCUSSION

Inflammation is the body’s immune response to infection, injury, insult or any other acute and chronic factors [41]. Immune cells and inflammatory cytokines such as tumor necrosis factor (TNF), tumor growth factor and interleukin (IL) are commonly involved in the process of inflammation [41]. The continuous presence of these inflammatory cytokines may alter the tissue microenvironment and cause mutation of normal cells. In addition, increasing attention has been paid to the role of immune cells and inflammatory cytokines in cancer etiology and progression [42–44]. Several immune-based therapies have been proposed to treat different cancers in clinical practice [45, 46]. More researchers have recognized the importance of inflammatory cytokines and immune cells in epithelial cancers [41], especially in mesenchymal stem cell-derived tumors such as OS [27, 47].

Multiple factors are involved in the etiology of OS, among which genetics and gene polymorphisms have aroused increased attention [1, 4, 8]. Given the important roles of inflammation and gene polymorphisms in cancer etiology and progression, more studies have been performed to explore the associations between inflammatory gene polymorphisms and susceptibility to OS [26, 29, 32, 33, 36–40, 48, 49]. Although several studies including Patio-Garcia et al. [36], Oliveira et al. [26], Zhao et al. [29] and Xie et al. [37] have explored the possible association between TNF-α 308G/A polymorphism and OS risk, there results are inconsistent. Other TNF polymorphisms have also been genotyped in OS patients *vs.* healthy subjects in an attempt to define the association of these polymorphisms with OS risk [26, 29, 36, 37]. Although a previous meta-analysis [30] had investigated the role of TNF-α 308G/A and TNF-β 252A/G polymorphisms in OS risk, it had the following demerits: 1) it only included TNF-α 308G/A, TNF-β 252A/G and TGF-β1 29T/C polymorphisms, devoid of other inflammatory gene polymorphisms such as IL-6 and IL-10; 2) it did not include the results reported by Zhao et al. [29], thus weakening its statistical power; 3) it did not discuss the impact of different ethnicities on the obtained result, knowing that ethnicity is an important factor affecting the association between gene polymorphisms and the disease. In addition, the results about IL-6 174G/C polymorphism are inconsistent between the study of Oliveira et al. [26] and that of Qi et al. [32], and the results about IL-10 1082A/G polymorphism are inconsistent between the study of Oliveira [26] and that of Cui et al. [33]. To the best of our knowledge, this is the first meta-analysis reporting the role of IL-6 174G/C and IL-10 1082A/G polymorphism in the etiology and progression of OS. This is also the first study reporting the association of TNF-β 252A/G, IL-6 174G/C and IL-10 1082A/G polymorphisms with OS, and the impact of ethnicity on OS susceptibility. Compared with previous meta-analyses, we retrieved more articles [29, 32, 33, 40] to explore the association of inflammatory gene polymorphisms with OS susceptibility.

### TNF-α 308G/A polymorphism and OS risk

TNF-α 308G/A polymorphism is a change G-to-A single nucleotide polymorphism (SNP) at position 308, affecting TNF-α gene regulation and associated with altered transcriptional activity in many diseases [50]. It has been found to play an important role in the etiology of many diseases such as systemic lupus erythematosus (SLE) [50], coronary artery disease (CAD) [51], and inflammatory bowel disease (IBD) [52]. The association between TNF-α 308G/A polymorphism and OS susceptibility has also been explored by many researchers. The results of studies performed by Oliveira et al. [26] and Patio-Garcia et al. [36] in Caucasian populations showed no significant association between them, while Zhao et al. [29] reported controversial results in Asian populations. Given these conflicting results, we performed this meta-analysis to determine whether TNF-α 308G/A polymorphism was truly associated with OS susceptibility in both Caucasian and Asian populations. Four relevant studies [26, 29, 36, 37] were included in this meta-analysis and the results showed that TNF-α 308G/A polymorphism was associated with an increased OS risk in the overall populations. Our study provided relatively objective results regarding the role of TNF-α 308G/A polymorphism and OS risk. Although these results are still inconsistent with the result reported by Bian et al. [30], they seem more creditable because they were retrieved from more studies. We believe that the most important factor contributing to this difference is the number of studies involved in our research. Our study was based on four eligible studies including Zhao’s study [29], which was not included in Bian’s study [30]. Another advantage of our study over Bian’s [30] is the sub-group analysis on ethnicity. As no sub-group analysis was performed in Bian’s study [30], their result only represents the overall population and cannot be used in either Caucasian or Asian population. In addition, no association was observed in either Asian or Caucasion population in sub-group analysis, although contradictory results were found in the overall populations. The combination of different original data in each study might have great impact on the pooled distribution of each genotype, which might be an important contributor to the different results of overall populations and Asians and Caucasians.

### TNF-α 238G/A polymorphism and OS risk

Another common SNP of TNF-α is TNF-α 238G/A polymorphism. It is recently believed that the A-238 allele at position 238 can significantly reduce the TNF-α production; on the other side, it may be associated with down-regulation of tissue inflammation [53]. Interestingly, we found that TNF-α 238G/A polymorphism played a protective role in the etiology of OS in Caucasian populations. Multiple factors are believed to be involved in the etiology of cancers or tumors, including gene, gene polymorphism, immune system, tissue microenvironment and eating habit. Inflammatory cytokines such as TNF-α occupy much of the tumor microenvironment, fostering tumor cell proliferation, survival and migration [23–25]. Therefore, inflammatory cytokines are considered important stimulators or factors contributing to many types of cancer and sarcoma. According to this consensus, it is easy to understand why TNF-α 238G/A polymorphism could significantly decrease the susceptibility to OS in Caucasian populations: A-238 allele of TNF-α 238G/A could significantly reduce the TNF-α production, and then it would indirectly reduce the immune response and change the immune microenvironment in tissues, thus reducing OS risk in Caucasian populations. However, the exact mechanism about how TNF-α 238G/A polymorphism affects TNF-α function and expression and plays a protective role in OS risk is not fully understood and needs further exploration [36]. In addition, there is no significant association between TNF-α 238G/A polymorphism and OS risk in either the overall population and Asian population, which further demonstrates the different roles of gene polymorphisms in various ethnicities. We could think that TNF-α 238G/A polymorphism might play different roles in the etiology of OS in different ethnicities as our Altman and Bland test showed that there was good evidence to support a different effect in Asians and Caucasians. However, what we should not ignore is that the limited number of included studies or the small number of included cases in individual studies might also contribute to this unremarkable relationship in Asian populations, which might not have enough statistical power to find out this difference.

### TNF-β 252A/G polymorphism and OS risk

TNF-β is a sub-type of the TNF family and believed to be involved in a variety of inflammatory, immune-stimulatory and antiviral responses [54]. Meanwhile, it is generally accepted that a SNP at position 252 in the intron of TNF-β called TNF-β 252A/G polymorphism leads to the overexpression of TNF-β, and it is considered a candidate biomarker of gastric cancer susceptibility [54]. Due to the function of TNF-β 252A/G polymorphism in many diseases [54–56], the relationship between TNF-β 252A/G polymorphism and OS susceptibility has also been determined [26, 37]. Oliveira ID et al. [26] studied TNF-β 252A/G polymorphism in 80 osteosarcoma patients and 160 control individuals using polymerase chain reaction-restriction fragment length polymorphism method. Although no remarkable relationship was observed in their study, they found that the Caucasian patients with a variant genotype (GG) of the 252A/G TNF-β gene showed an event-free survival rate of 20% at 100 months. In their opinion, the high-dose local TNF management selectively destroyed tumor blood vessels and had powerful anticancer action, but when chronically produced these cytokines might act as an endogenous tumor promoter, contributing to the tissue remodeling and stromal development necessary for tumor growth and spread [26]. This may be the reason why TNF-β 252A/G polymorphism had impact on the event-free survival rate. However, no significant association of TNF-β 252A/G polymorphism with the survival rate was found in Asian populations [37]. The inconsistent backgrounds, the relatively small sample size and different genotyping technologies might be important contributors to this difference. Our study showed that TNF-β 252A/G polymorphism was not significantly associated with OS susceptibility in either overall populations, Asian populations or Caucasian populations, which is consistent with Oliveira’s study [26] and Xie’s study [37]. However, whether TNF-β 252A/G polymorphism affects the survival rate in Asian populations remains unknown and needs further investigation.

### TGF-β1 29T/C polymorphism and OS risk

Three studies [38–40] were performed to detect the relationship between TGF-β1 29T/C polymorphism and OS susceptibility, hoping to find the important roles of TGF-β1 29T/C polymorphism in the etiology of OS, knowing that this cytokine plays a key role in cancer etiology [57]. TGF-β1 is known to inhibit cellular proliferation or promote cellular differentiation and apoptosis in normal cells [57]. However, like TNF, the expression of TGF-β1 appears to be increased in cancer cells [57]. Wu et al. [40] reported that patients carrying TT genotype had a higher risk to get OS than patients carrying CC genotype, and that the morbidity and metastasis of OS were relevant to TGF-β1 29T/C polymorphism. These results are consistent with Xu et al. [39], who reported that the prevalence of TT genotype and T allele was significantly elevated in patients as compared with healthy controls. According to Ma et al. [38], the TT genotype among 29TC polymorphism of TGF-β1 gene might also be a genetic factor that aroused OS. However, no significant association was observed between TGF-β1 29T/C polymorphism and OS susceptibility. Our meta-analysis recruited the original data of Wu et al. [40], Xu et al. [39] and Ma et al. [38], based on which we performed a pooled analysis. When all the data were merged, the distribution of TT, CT and CC genotypes might be changed among OS patients and healthy subjects, which might be the most important contributor to the different results of our meta-analysis and the above three studies. Although the meta-analysis of Bian et al. [30] found the same result as that of the above three studies, our study might be more creditable than theirs, as we included more studies with larger sample sizes. In addition, all the studies included in our meta-analysis were performed in Asian populations, and whether TGF-β1 29T/C polymorphism increases or decreases OS susceptibility in Caucasian populations remains unclear and more studies are required.

### IL-6 174 G/C and IL-10 1082A/G polymorphisms and OS risk

Interleukins are important members of inflammatory cytokines that have been regarded as key contributors to tumor development with respect to proliferation, apoptosis, angiogenesis and differentiation [32, 33, 58, 59]. IL-6 and IL-10 are the most commonly studied interleukins and always studied together in many studies, knowing that both IL-6 and IL-10 are pro-inflammatory cytokines that may enhance the pro-inflammatory status in different physiologic and pathophysiologic processes. It has been well documented that IL-6 and IL-10 polymorphisms play a key role in many diseases [58, 60–62]. Many studies have also been conducted to explore the association between IL-6 and IL-10 polymorphisms and OS [26, 32, 33]. Oliveira [26] performed the first study to determinate whether there existed difference in the distribution of IL-6 and IL-10 polymorphisms between OS patients and health controls but found no remarkable correlation in Caucasian populations. In contrast, Qi et al. [32] reported that the genotype CC of IL-6 174 G/C carried a higher risk of OS metastasis and later Enneking stages, compared with the GG genotype. They thought that the IL-6 −174 G/C genotype was associated with the risk for OS development and metastasis in Chinese Han population. The ethnicity, sample size, selection bias of cases and control subjects, and genotyping technologies might contribute to this difference. Our pooled results showed that there was no significant association between IL-6 174 G/C polymorphism and OS susceptibility in Caucasian populations, and the controversial results were observed in Asian population, which is consistent with Oliveira et al.’s [26] and Qi et al.’s study [32]. These controversial results might be attributed to the different genetic backgrounds in Asians and Caucasians because the results of Altman and Bland test in our study showed that there was good evidence to support a different effect in Asians and Caucasians, while we could not ignore the influences of other factors such as the limited number of included studies or the small number of included cases in individual studies. In addition, we also found an insignificant association in the overall populations, which might result from the changed gene distributions in the pooled analysis. As to IL-10 1082A/G polymorphism, two studies were included in our meta-analysis [26, 33]. In the overall populations, our meta-analysis showed a significant association between 1082A/G polymorphism and OS risk, indicating that the IL-10 1082G allele might be the genetic risk and susceptibility factor for developing OS. It has been verified that the 1082A allele could down-regulate the IL-10 transcription, whereas the IL-10 1082G allele has an increasing effect on IL-10 expression [63]. Moreover, IL-10 has impact on pro-inflammatory cytokine gene transcription and stability by reducing protein translation, which might be an important contributor to the etiology and development of many diseases [63]. Therefore, it is easy to understand why the IL-10 1082G allele in OS patients significantly differed from that in the control group. Besides, Cui et al. [33] found that there was also a statistically significant association between the IL-10 1082A/G genotype and poor survival, further verifying the role of 1082G allele in the etiology and development of OS and survival of OS patients. Our sub-group analysis revealed a remarkable relationship between IL-10 1082A/G polymorphism and OS risk in Asian populations, which is consistent with Cui’s study [33], while the IL-10 1082G allele might not have significant impact on the etiology of OS in Caucasian populations. However, we could not only consider the different role of IL-10 1082A/G in OS etiology as the contributor to the contrast results in Asian and Caucasian populations, as our Altman and Bland test showed that there was no good evidence to support a different effect in Asians and Caucasians. Other factors, such as the limited number of included studies, the small number of included cases in individual studies, and different genotyping techniques might also have great impact. Therefore, the effect of 1082A/G polymorphism on OS in different ethnicities needs to be further explored.

Despite the comprehensive analysis on the association between inflammatory gene polymorphisms and OS, our meta-analysis still has some limitations. First, of the nine studies that eventually satisfied the eligibility criteria of the present study, only two studies were included in the pooled analysis of TNF-a 238G/A, TNF-β 252A/G, IL-6 174G/C, and IL-10 1082A/G polymorphisms. Second, the sample size of some studies used in this meta-analysis is still relatively small, which might contribute to the inconsistent results and affect the conclusions drawn. Third, no study was conducted to explore the association between TGF-β1 29T/C polymorphism in Caucasian populations, which may not be sufficient to make a convincing conclusion on all ethnicities and indicate a race-specific effect. Therefore, larger-scale and better-designed studies are necessary to determine the association between inflammatory gene polymorphisms (TNF-α 308G/A, TNF-α 238G/A, TNF-β 252A/G, TGF-β1 29T/C, IL-6 174G/C and IL-10 1082A/G) and OS susceptibility.

## CONCLUSIONS

In summary, this is the first meta-analysis to determinate the association of TNF-α 308G/A, TNF-α 238G/A, TNF-β 252A/G, TGF-β1 29T/C, IL-6 174G/C and IL-10 1082A/G polymorphisms with OS susceptibility. Inflammatory gene polymorphisms play a key role in the occurrence and progression of OS. IL-6 174G/C polymorphism was obviously associated with OS risk in Asian populations, while TNF-α 238G/A polymorphism might be associated with the decreased susceptibility to OS in Caucasians. Although controversial associations were observed between IL-10 1082A/G and OS risk in Asian and Caucasian populations, it is difficult to make a definite conclusion about the role of IL-10 1082A/G polymorphism in the etiology of OS because our Altman and Bland test showed that there was no good evidence to support a different effect in Asians and Caucasians.
